# Visual Odometry Based on Structural Matching of Local Invariant Features Using Stereo Camera Sensor

**DOI:** 10.3390/s110707262

**Published:** 2011-07-18

**Authors:** Pedro Núñez, Ricardo Vázquez-Martín, Antonio Bandera

**Affiliations:** 1 Departamento de Tecnología de los Computadores y las Comunicaciones, University of Extremadura, Escuela Politécnica, Avda. Universidad s/n, 10071 Cáceres, Spain; 2 CITIC Centro Andaluz de Innovación y Tecnologías de la Información y las Comunicaciones, Parque Tecnológico de Andalucía, 29590 Málaga, Spain; E-Mail: rvmartin@uma.es; 3 Departamento de Tecnología Electrónica, University of Málaga, E.T.S.I. Telecomunicación, Campus Teatinos 29071 Málaga, Spain; E-Mail: ajbandera@uma.es

**Keywords:** visual odometry sensor, stereo vision sensor, robotic, combined constraint matching algorithm, maximum-weighted clique

## Abstract

This paper describes a novel sensor system to estimate the motion of a stereo camera. Local invariant image features are matched between pairs of frames and linked into image trajectories at video rate, providing the so-called visual odometry, *i.e.*, motion estimates from visual input alone. Our proposal conducts two matching sessions: the first one between sets of features associated to the images of the stereo pairs and the second one between sets of features associated to consecutive frames. With respect to previously proposed approaches, the main novelty of this proposal is that both matching algorithms are conducted by means of a fast matching algorithm which combines absolute and relative feature constraints. Finding the largest-valued set of mutually consistent matches is equivalent to finding the maximum-weighted clique on a graph. The stereo matching allows to represent the scene view as a graph which emerge from the features of the accepted clique. On the other hand, the frame-to-frame matching defines a graph whose vertices are features in 3D space. The efficiency of the approach is increased by minimizing the geometric and algebraic errors to estimate the final displacement of the stereo camera between consecutive acquired frames. The proposed approach has been tested for mobile robotics navigation purposes in real environments and using different features. Experimental results demonstrate the performance of the proposal, which could be applied in both industrial and service robot fields.

## 1. Introduction

In order to accomplish higher-level tasks, autonomous mobile robots must typically be able to determine their pose (position and orientation) while moving. To address this problem, absolute localization approaches usually employ the estimation of the robot’s displacement in the environment between consecutively acquired perceptions as one of their inputs. Typically, this relative localization or pose tracking is performed using wheel odometry (from joint encoders) or inertial sensing (gyroscopes and accelerometers). However, wheel odometry techniques cannot be applied to robots with non-standard locomotion methods, such as legged robots. Besides, it suffers from precision problems, since wheels tend to slip and slide on the floor [1]. On the other hand, inertial sensors are prone to drift. Vision is an alternative to these systems which have acquired growing importance in the mobile robotics community due to their low cost and the information they can provide compared to other robotic sensors. In robotics and computer vision, visual odometry defines the process of estimating the pose of a robot by analyzing the images provided by the camera(s) mounted on it. As other visual-based techniques, this issue has come into vogue in these last years. Thus, Nistér *et al.* [2] proposed an approach to estimate the motion of a stereo pair or single camera in real-time. This approach employs Harris corners and uses normalized correlation over an 11 × 11 window to evaluate potential matches. Konolige and Agrawal [3] describes a frame-frame matching in real time to estimate the 3D egomotion and use this estimate for visual Mapping. Similar work is presented by Klein *et al.* [4], which is applied for the SLAM problem. The MER’s visual odometry (MER-VO) [5] also uses a corner detector and a pseudo-normalized correlation to determine the best match. It uses the on-board position from wheel odometry as an initial estimate to decrease run time. With the aim of tracking a large number of features and still not relying on this initial estimate, the MER-VO has been improved [6]. The visual odometry implemented for the Mars Science Laboratory (MSL) mission is at least four times more computationally efficient than the MER-VO, but it follows similar guidelines. These approaches perform a feature-based stereo matching as a preliminary stage.

The matching process represents a crucial step for an accurate visual odometry sensor. In fact, it constitutes the main hurdle to overcome in order to achieve a robust approach. In the Nistér’s proposal [2], corners are matched between consecutive pairs of frames. To obtain the set of accepted matches both in stereo and in video, all features which are a certain disparity limit from each other are matched. Only pairs of corners which mutually have each other as the preferred mate are accepted as valid matches. This algorithm assumes very small robot displacement between frames. The approach from Pretto *et al.* [7] employs a similar strategy to estimate the relative camera motion from two calibrated views, but it matches interest points between pairs of frames using the Best Bin First (BBF) algorithm. This strategy is described as a feature tracking [8]: features are selected and located in the subsequent frame using spatial correlation search. The MER-VO and MSL-VO also rely on feature tracking. Other approaches use feature matching rather than tracking [9]. In these approaches, features are selected and then matched based on a descriptor associated with the feature. These approaches do not necessarily require an initial motion estimate, but they require salient detectors and stable descriptors to work well with large robot motions. The Hirchmuller’s [9] and Howard’s [8] approaches employ a stereo range data for inlier detection.

**Figure 1.**
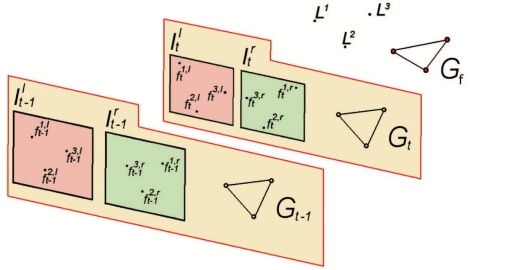
Problem statement: given the pairs of stereo images taken at frames *t* − 1 and *t*, the robot motion is estimated from the natural landmarks {*L*}*^i^*. Two graphs emerge from the stereo and feature matching stages.

This paper proposes a visual odometry system which consists of two consecutive feature matching stages (see Figure 1). The first stage matches points of interest obtained from the left and right images, achieving stereo matching. This matching will be constrained by the stereo geometry—matched points must be in the same epipolar line—and considering the feature descriptors. Taken into account these constraints, a consistency matrix is computed for all pairwise combinations of matches. Weights are assigned to the non-zero elements of this matrix as a function of the distance between the computed descriptors of the matched features. These weights are inversely proportional to the distance between descriptors, *i.e.*, they increase when the distance between descriptors decreases. This matrix is used to find the largest-valued set of mutually consistent matches. This is equivalent to finding the maximum-weighted clique on a graph defined by this adjacency matrix. The aim is to provide a set of features which will be defined by their 3D world positions in the camera coordinate system. These features are considered as natural landmarks in the environment and they emerge from the scene as a graph, not as individual items. Then, the second stage performs matching between sets of natural landmarks associated to consecutively acquired pairs of stereo images. This matching will be also constrained by the relative distance between the positions of the 3D features and the computed difference between their descriptors. This second matching stage is also stated as a maximum-weighted clique problem. This last stage allows to track the robot pose using an Absolute Orientation (AO) technique and minimizing not only the algebraic error, but also the geometric error [10].

This approach is very related to the works of Hirchmuller [9] and Howard [8]. However, contrary to these approaches, we do not employ a dense disparity map computed by a separate stereo algorithm. When computing resources are limited, generating this dense map could be undesirable [6]. Besides, these approaches usually need images with textures. On the other hand, Howard’s approach employs a corner detector and uses the sum-of-absolute differences (SAD) between feature descriptors to compute the score matrix for all pairwise combinations of features in both feature sets. In our experiments, we will employ different detectors and descriptors. Scale-invariant features, such as the SIFT [11], will allow to match features although the robot does not move a small distance between subsequently acquired frames. However, the invariance against rotation and scale change is computationally very costly with SIFT. When significant scale changes and rotations around the optical axis is not present, other descriptor like the Speeded Up Robust Features (SURF) [12] or corner-like image features, has been chosen and tested (see Section 3). Finally, whereas the Howard’s work uses a maximum clique algorithm to obtain a structural consistent feature matching, this paper proposes to search for a maximum-weighted clique.

The paper is organized as follows: Section 2 describes the proposed approach for stereo visual odometry. Experimental results and a comparison of the proposed approach with other related methods are presented in Section 3. Finally, the main conclusions and future work are drawn in Section 4.

## 2. Proposed Approach for Stereo Visual Odometry

**Figure 2.**
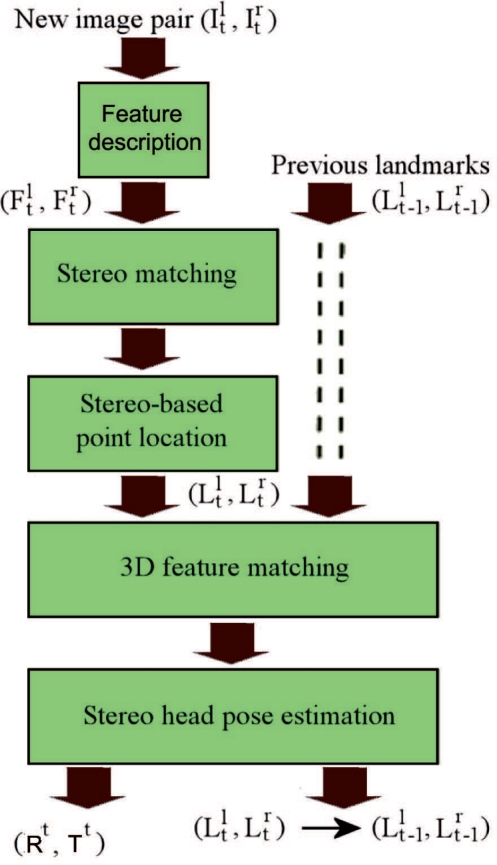
Overview of the proposed visual odometry approach.

The aim of the visual odometry sensor is to calculate an estimate of each 6DOF (degree of freedom) robot pose, with translation *T^t^* and rotation *R^t^* in the *t^th^* frame. In the proposed approach, two consecutive image pairs acquired by the stereo cameras mounted on the robot are matched to estimate the displacement of the mobile platform. The quality of this matching process is crucial to obtain an accurate estimation. Thus, a significant advance in visual odometry algorithms is the possibility of improving the matching process using consecutive stages [8]. Our proposal follows this scheme, whose block diagram is illustrated in Figure 2. As shown in the figure, the proposed visual odometry algorithm consists of two matching processes performed in five steps. Firstly, each new image pair is acquired and two sets of points of interest and their associated descriptors are obtained. Both sets of features are the input of the next step, which computes the stereo matching. A robust matching is achieved by building a consistency matrix for all pairwise combinations of tentative matchings. Then, the algorithm finds the largest-valued set of mutually consistent matchings by looking for the maximum-weighted clique on the graph with adjacency matrix equal to the computed consistency matrix. The 3D locations of these natural landmarks in the environment are calculated in the third step using the output of the stereo matching process. Next, the 3D landmark association step performs matching between the sets of features which belong to consecutively acquired stereo images. The output of this step is employed to estimate the robot displacement at current instant of time. Each one of these steps is explained in details in the next Sections.

### 2.1. Local Invariant Image Features

**Figure 3.**
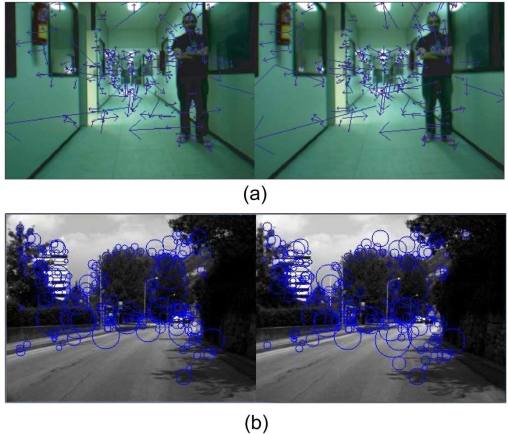
(**a**) SIFT features found for the left and right images from the stereo image (*F^l^_t_* and *F^r^_t_*). The scale and orientation are indicated by the size and orientation of the vectors; (**b**) SURF features calculated using the stereo system in an outdoor environment. Scale are illustrated by the size of the circles (orientation is not shown in the figure).

Local features are image patterns that differ from its immediate surroundings. They are typically associated to changes of image properties. Let *I^r^_t_* and *I^l^_t_* be the right and left images captured using the stereo camera at time *t*. This first step detects the set of features in the left and right images, *F^l^_t_* and *F^r^_t_*, respectively. As it will show in Section 3 we have tested different feature detectors and descriptors, like corner-like image features (Harris detector [13] and a simple descriptor associated to the corners based on the correlation window of the neighborhood), SIFT and SURF (see Figure 3(a, b)). These features are associated to vectors which represent the location (*x*, *y*) and other properties associated to the particular descriptor, like scale and orientation (see Figure 3). Depending on the final application, like robot speed, environment, type of robot (e.g., wheel or legged robots), it would be better to choose a specific pair of detector/descriptor.

### 2.2. Stereo Matching and Stereo-Based Point Location

In this section, we formulate the stereo matching problem as a graph-theoretic data association problem. The main advantage of our method with respect to other stereo matching approaches is its robustness in the data association stage, which will finally improve the ego-estimation of the robot motion. This stereo matching does not provide a dense depth map, which is not necessary for us since our proposal deals not with mapping but only with relative localization. Contrary to other related approaches [8], our aim is to deal with good individual matchings, avoiding failures due to scenarios where a dense stereo map cannot be correctly obtained.

**Figure 4.**
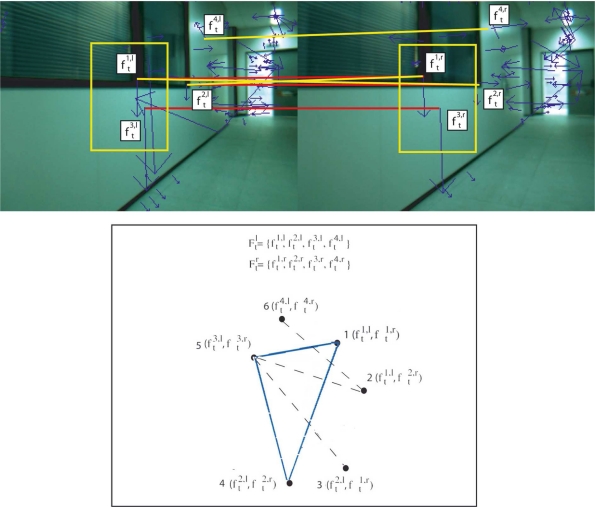
Vertices represent tentative matchings when considered individually. Arcs indicate compatible associations, and a clique is a set of mutually consistent associations (e.g., the clique {1, 5, 4} implies that associations *f*^1,^*^l^*_t_ → *f*^1,^*^r^_t_*, *f*^2,^*^l^_t_* → *f*^2,^*^r^_t_*, *f*^3,^*^l^_t_* → *f*^3,^*^r^_t_* may coexist).

**Figure 5.**
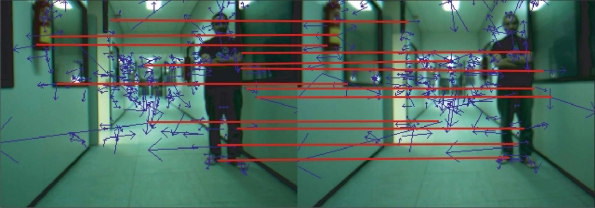
Matched SIFT features between left and right images from the stereo pair shown in Figure 3. Red line represents matched points.

The fundamental data structure of this step is the correspondence graph [14], which represents valid associations between the two sets of feature descriptors (see Figure 4). Complete subgraphs or cliques within the graph indicate mutual associations compatibility and, by performing a maximum-weight clique search, the joint compatible association set emanated from the better matchings of descriptors may be found. Construction of the correspondence graph is performed through the application of relative and absolute constraints. Thus, vertices of the graph indicate individual association compatibility and are determined by absolute constraint. On the other hand, the arcs of the correspondence graph indicate joint compatibility of the connected vertices and are determined by relative constraints. The weight associated to each vertex is related to the similarity measure of corresponding descriptors. The method used to calculate the correspondence graph has three major stages:

1. *Definition of the vertices of the correspondence graph*. In the proposed method, graph vertices are associated to tentative matchings of features from *F^l^_t_* and *F^r^_t_* after applying an absolute constraint. Let |*F^l^_t_*| and |*F^r^_t_*| be the number of feature descriptors for left and right images, respectively. Firstly, the algorithm generates the matrix *T_t_* (|*F^l^_t_*| × |*F^r^_t_*|) for all pairwise combinations calculating the Euclidean distance between their associated descriptors. Therefore, the matrix item associated to the matching of two similar features presents a low value. On the other hand, high values at *T_t_* correspond to dissimilar features. Besides, this matrix is modified at the same time to satisfy some of the constraints described in Se *et al.* [15] (epipolar, disparity, unique match constraints, and, if these parameters are available, orientation and scale). Pairwise matched features whose matrix values are lower than a fixed threshold $U_{T}^{t}$ constitute the set of tentative matchings. Thus, graph vertices are defined as the set of all possible combinations of these pairwise descriptors (e.g., vertex 1 in Figure 4 is valid if descriptor *F*^1,^*^l^_t_* is a possible correspondence of *F*^1,^*^r^_t_*). A weight array whose items are equal to the inverse of the tentative matchings of *T_t_* is also stored. These weights will be used to find the largest-valued set of mutually consistent matches.
2. *Definition of the arcs of the correspondence graph*. For all pairwise combinations of matchings in *T_t_*, a relative constraint matrix is calculated, *R_t_*. To do that, a relative constraint on the image coordinates is used. This relative constraint takes into account feature parameters that allow to reference one feature with respect to the other. For instance, if SIFT descriptors are used, the vector will be defined by *ω* = (*o*, *s*)*^T^*, where *o* and *s* are the orientation and scale values associated to the descriptor. In this particular case, a pair of matched descriptors is consistent if the Euclidean distance between the *ω* vectors from two SIFT descriptors in the left image is similar to the Euclidean distance between the corresponding vector in the right image. That is, a pair of matches (*f^i^*^,^*^l^_t_*, *f^i^*^,^*^r^_t_*) and (*f^j^*^,^*^l^_t_*, *f^j^*^,^*^r^_t_*) are consistent iff they satisfy the relative constraint:
  (1) $$\left\|\omega_{t}^{l}-\omega_{t}^{r}\right\|\leq U_{R}^{t},$$
  being
  (2) $$\begin{array}{l} \omega_{t}^{l}=\sqrt{{\left(o_{t}^{i,l}-o_{t}^{j,l}\right)}^{2}+{\left(s_{t}^{i,l}-s_{t}^{j,l}\right)}^{2}}\\ \omega_{t}^{r}=\sqrt{{\left(o_{t}^{i,r}-o_{t}^{j,r}\right)}^{2}+{\left(s_{t}^{i,r}-s_{t}^{j,r}\right)}^{2}} \end{array}$$
  where (*o*, *s*)*_i_* and (*o*, *s*)*_j_* denote the orientation and scale values of a SIFT descriptor and $U_{R}^{t}$ is a threshold defined by the user. Thus, the corresponding entry in the relative constraint matrix *R_t_* contains a 1 value if the constraint is satisfied (arc in the graph), and 0 otherwise. For instance, in Figure 4, the relative constraint between (*f*^1,^*^l^_t_*, *f*^3,^*^l^_t_*) and (*f*^1,^*^r^_t_*, *f*^3,^*^r^_t_*) matches, and then vertex 1 is connected to vertex 5. On the contrary, the relative constraint between (*f*^4,^*^l^_t_*, *f*^3,^*^l^_t_*) and (*f*^4,^*^r^_t_*, *f*^3,^*^r^_t_*) does not match. Hence, vertices 6 and 5 are not connected.
3. *Maximum-weight clique detection.* The set of mutually consistent matches which provides a largest total weight is calculated. This is equivalent of finding the maximum-weight clique on a graph with adjacency matrix *R_t_*. Specifically, the approach to solve the maximum-weight clique problem implements the algorithm proposed by Kumlander [16]. This algorithm is based on the classical branch and bound technique, but employing the backstracking algorithm proposed by Ostergard [17] and a vertex-coloring process to define a more efficient pruning strategy. After applying the maximum-weight clique algorithm, this stage obtains a set of mutually compatible associations, that is, a set of matched features. In this way, the algorithm takes into account structural relationships to avoid bad associations, which could result in erroneous displacement estimates. Figure 5 shows the pairwise descriptors after using the proposed stereo matching algorithm. As it is illustrated in the figure, the quality of the matching process is guaranteed even though the number of features is high. In the example in this figure, the number of matched features was 21.

Each detected feature is readily characterized by the Cartesian localization of the point of interest provided by the stereoscopic vision system.

### 2.3. 3D Feature Association

**Figure 6.**
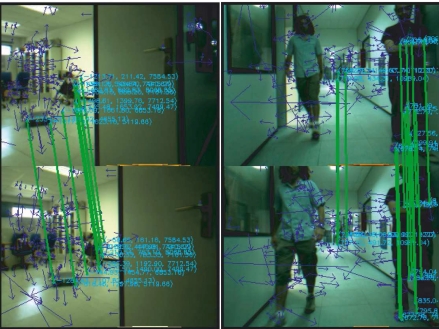
Feature association results for two different displacements. After applying the maximum-weighted clique algorithm the number of pairwise matched features is 7 and 13 for the left and right images, respectively (3D coordinates of the landmarks are also included).

Let $I_{t-1}^{l,r}$ and $I_{t}^{l,r}$ represent the pairs of stereo images taken with the robot camera at two consecutive intervals of time. For each pair of images, the approach detects the points of interest and computes their descriptors, performing the stereo matching as it is described in Section 2.2. This process will provide two sets of natural landmarks, *L_t_*_−1_ and *L_t_*. Then, the proposed approach performs the 3D feature matching using the same data association technique described in Section 2.2, that is, the correspondence problem is achieved between the two sets of 3D features applying absolute and relative constraints. Firstly, a measure distance between feature descriptors is used to obtain the matrix *T_F_*. Thus, entries in *T_F_* whose value are lower than a fixed threshold $U_{T}^{f}$ constitute the set of tentative matchings. The inverse of these values are stored in a weight array. Next, the relative constraint is used to generate the adjacency matrix *R_f_* from the set of possible pairwise landmarks. Similar to the stereo matching stage, this relative constraint takes into account features parameters that will allow to reference one landmark with respect to the other. Thus the relative constraint associated to the location of each pair of landmarks, ( $L_{t-1}^{i}$, $L_{t-1}^{j}$) and ( $L_{t}^{i}$, $L_{t}^{j}$), is used:

(3) $$\left\|L_{t-1}^{i}-L_{t-1}^{j}\right\|-\left\|L_{t}^{i}-L_{t}^{j}\right\|\leq U_{R}^{f}$$

where $\left\|L_{t}^{i}-L_{t}^{j}\right\|$ is the Euclidean distance between landmark locations and $U_{R}^{f}$ is an user-defined threshold. Finally, the maximum-weight clique algorithm is applied to the adjacency matrix *R_F_* and the set of mutually consistent matchings is computed. Figure 6 illustrates the feature association between two consecutive frames *t* − 1 and *t*. The output of this stage provides a set of accurate pairwise matched features, which are used to obtain the displacement estimate.

### 2.4. Stereo Head Pose Estimation

The purpose of the two-stages matching process described in previous Sections is to provide a set of 3D landmark matchings between consecutive frames. Let *M* denote the set of *N_M_* 3D landmark matchings, $M={\left\{(m_{t-1}^{i},m_{t}^{i})\right\}}_{i=1:N_{M}}$. This set will allow to estimate the robot’s displacement between two consecutive acquired frames. In the related literature, this problem is typically accomplished by means of absolute orientation techniques. The solution of this problem consists of minimizing the error function

(4) $$E(R^{t},T^{t})=\sum_{i=1}^{N_{M}}{\sum_{j=1}^{N_{M}}\eta_{ij}\left\|m_{t-1}^{i}-(R_{\Delta \theta m_{t}^{j}}+\Delta T)\right\|}^{2}$$

where $m_{t-1}^{i}$ and $m_{t}^{j}$ are matched landmarks belonging to *M*, *η_ij_* is a binary value defined as 1 if $m_{t-1}^{i}$ and $m_{t}^{j}$ have been matched or 0 otherwise, and *R^t^* and *T^t^* are the rotation and translation matrices whose values are sought. As it was shown in [18], SVD decomposition and quaternion techniques produce the best results. In this work, we use the well-known SVD technique described in [19]. This method estimates the 6DOF robot pose decoupling the parameters by centering each of the points sets about their centroids. However, this computation of motion minimizes an error on the 3D feature location (algebraic error). It produces a permanent motion bias. In order to reduce it, an image based error (*i.e.*, geometric error) should be minimized [10]. Thus, the previous result based on the SVD technique is used as initial estimate, *T*_0_, of the iterative process for minimizing this geometric error. Nonlinear LSE optimization (Gauss–Newton), starting from this initial guess *T*_0_ in order to ensure convergence, is used for estimating the final robot pose [10].

## 3. Experimental Results

In this section, the proposed visual odometry sensor has been analyzed. The main novelty of this work, the combined constraint matching algorithm which includes the search for the maximum-weight clique on the graphs, is evaluated in terms of robustness and computational load for different descriptors, and it is compared with other three feature matching approaches. Thus, results of the proposed approach are compared against (i) a matching algorithm based on the geometric transformation model [20] (RANSAC + epipolar geometry), (ii) the Best-bin-first (BBF) search method proposed by Beis and Lowe [21], which is a modification of the k-d tree algorithm, and (iii) the matching approach also based on the combined constraint algorithm which uses the search for the maximum clique described in our previous work [22].

Feature matching accuracy is very important and depends on the feature types. Choice of algorithms to extract features and descriptors depends on the environment and application. In order to evaluate the proposed Visual Odometry method, different detectors and descriptors have been used in different real scenarios: corner-like image features (Harris corners [13]), faster but less stables, and SIFT [11] or SURF [12], more stables but higher computational load. Typically, the major problem of the SIFT feature detector is the long time taken to extract the features from the images when compared to other approaches. Implementation of SIFT for GPU (SiftGPU) [23] has been used in this paper as a previous stage to detect features from the stereo image pair. The corner-like image descriptor is based on the correlation window of the neighborhood surrounding the Harris corners. Rest of the methods have been implemented in C++. To compare the proposed approach against our previous work, the same parameters employed to build the emerged graphs have been used. Finally, we describe a set of experiments conducted in real robot environments (indoor and outdoor) to demonstrate the validity of the visual odometry sensor. These scenarios include dynamic elements (e.g., persons), occlusions, ambiguities and situations where the robot closes a loop while moving. Besides, in order to validate our results, the robot was moved in a closed loop on a typical indoor environment, calculating the error between the start and end poses.

Previously, to properly evaluate the matching stages, it is necessary to carry out a correct selection of a set of parameters. Specifically, these parameters are associated to thresholds in the graph emerging stages. Next subsection explains the method used for estimating these parameters. Then, Sections 3.2 and 3.3 describe the features matching algorithms and the visual odometry application, respectively.

### 3.1. Estimation of Parameters

Our approach needs to adjust a set of thresholds which determines the reliability of the composed graph. The values of these design parameters are associated to the absolute and relative constraints of the graph emerging steps. Therefore, these thresholds are described according to the matching stage in where they are used (*i.e.*, stereo or feature matching).

Stereo Matching Stage

- The $U_{T}^{t}$ threshold is related to the nodes of the graph *G_t_* for the stereo matching stage. Given two features, this parameter determines the higher value for being considered as pairwise matched features, according to absolute constraint (*i.e.*, the similarity of the descriptor or the epipolar constraints).
- The $U_{R}^{t}$ threshold is related to the arcs of the graph *G_t_*. This parameter evaluates the consistence of two nodes of the graph (two pairwise matched features) according to local constraints. In this stage, this relative constraint will depend on the feature type (e.g., the orientation and scale information associated to the descriptors or the distance of the features).

Feature Matching Stage

- The $U_{T}^{f}$ threshold is defined as the higher value for considering two landmarks acquired in different instants of time as candidate to be a correct match using absolute constraint (*i.e.*, the similarity of the descriptors). Similar to the stereo matching stage, this threshold is related to the nodes of the graph *G_f_* for the feature matching stage.
- The $U_{R}^{f}$ threshold is also related to the arcs of the graph *G_f_*, that is, its adjacency matrix. Given two pair of candidates for being real matched landmarks, $U_{R}^{f}$ is the higher value for determining their consistence according to local constraints (3D location of the features).

The benchmark performed to set them correctly has been similar for the two stages. This step is based on Blanco’s work [24]. For both descriptors, SIFT and SURF, optimal thresholds are calculated by minimizing the probability *P_err_* of misclassifying a association as a valid (*v*) or an invalid (*w*) candidate. It is described as:

(5) $$\begin{array}{lll} P_{err}(U_{T},U_{R}) & = & P(w)P_{err}(U_{T},U_{R}|w)+P(v)P_{err}(U_{T},U_{R}|v)\\ & = & P(w)P(d_{ij}<U_{T},\delta_{ij}<U_{R}|w)\\ & + & P(v)[1-P(d_{ij}<U_{T},\delta_{ij}<U_{R}|v)] \end{array}$$

Where a misclassification will occurs when: (i) a distance *d_ij_* is less than both thresholds *U_T_* and *U_R_*, and it was a wrong correspondence, or (ii) a valid pairing does not pass the thresholds *U_T_* and *U_R_*. Considering no a priori information about the probability of being in a valid or invalid association, that is *P* (*v*) = *P* (*w*) = 1/2, the method evaluates the joint conditional densities *p*(*d_ij_*, *δ_ij_*|*v*) and *p*(*d_ij_δ_ij_*|*w*) from histograms according to a set of 40 pairs of images with 10 landmarks for which is known the ground-truth (*i.e.*, its location in 3D space). Table 1 summarizes the thresholds for the minimum classification error *P_err_* for the SIFT and SURF descriptors.

### 3.2. Evaluation of the Robustness and Time Processing

**Figure 7.**
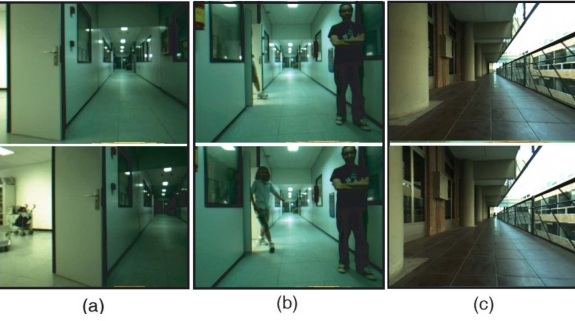
A set of 320 × 240 images acquired by the camera has been used to evaluate the robustness and time processing of the matching algorithm. **(a)** a camera movement (translation and rotation); **(b)** a significant change in the scene; and **(c)** ambiguities due to similar objects in the scene.

Robustness and computational load of the proposed matching algorithm have been evaluated and compared against three different matching methods: the BBF algorithm [21], the matching method based on the geometric transformation model [20] and the matching approach presented in our previous work [22]. To validate the approach, a set of images collected by a camera has been used. These images correspond to regular combinations of camera movements (e.g., rotation or translation), scenes where there is a significant change (e.g., dynamic object) and captures where there are significant ambiguities (e.g., similar objects). This set consists of 100 pairs of 320 × 240 images acquired in indoor and outdoor environments. Figure 7(a–c) show a representative selection for each case of study.

For each image, the SIFT features are computed [11] and matched using each particular matching method. Using this set of pairwise matched features, we have manually selected 50 correct matches of them, or the maximum number of correct matches, if there are less than 50 correct matches (this value is considered as *Total positives*). Next, incorrect pairwise matched features are randomly generated. These outliers are added to the positive set in increasing amounts, so that they are going to represent from 10% to 90% of the total resulting set in increments of 10%. Next, the matching algorithms are applied to the final set of matched features. For every percentage of outliers, this process is repeated 100 times (100 times × 100 images = 10,000 samples per each percentage of outliers).

**Figure 8.**
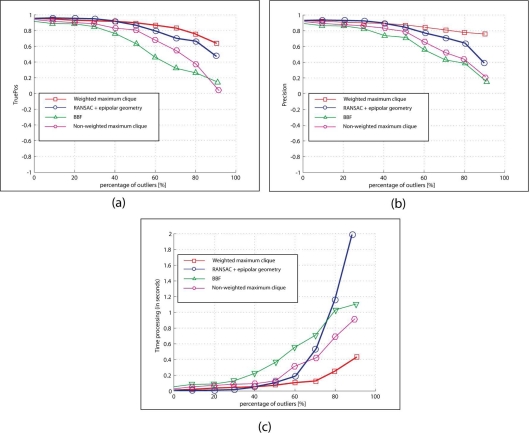
Performance of the matching algorithms used in the comparative study for various percentage of outliers. **(a)** True Positives against to different percentage of outliers; **(b)** Evolution of the precision against to different percentage of outliers; and **(c)** Time processing against the percentage of outliers. See the text for more details.

**Figure 9.**
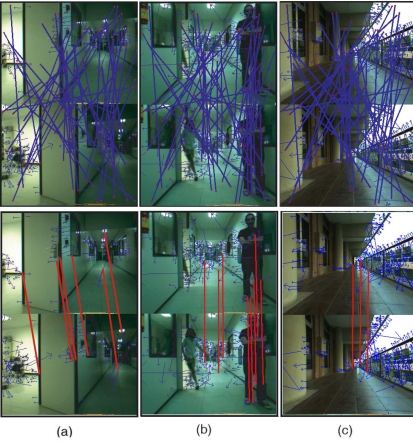
Illustrative examples of the matching algorithm proposed in our visual odometry system for three different image tests used in the comparative study (results of the matching process for the images of the Figure 7**(a–c)**, respectively). On the top, the initial matching which includes the 80% of outliers is shown. Below, results of the matching algorithm used in our approach have been drawn.

To evaluate the robustness of the matching algorithm which is included in the proposed visual odometry system, we defines the following measurements:

(6) $$\begin{array}{l} \text{TruePos}=\frac{\text{NumberTrueMatches}}{\text{Totalpositives}}\\ \text{Precision}=\frac{\text{NumberFalseMatches}}{\text{NumberFalseMatches}+\text{NumberFalseMatches}} \end{array}$$

where *Number True Matches* is the number of correct matches, *Number False Matches* is the number of incorrect matches, and *Total positives* is the number of correct matches selected at the beginning of the tests. The average performance of the matching methods after the total experiment is given in Figure 8 and summarized in Table 2. Figure 8(a) represents the evolution of the *TruePos* against the percentage of outliers. From this figure, it can be noted that the average *TruePos* value is high for each algorithm when the percentage of outliers is lower than 50%. After this value, due to the high number of outliers, the efficiency of the algorithms decreases. However, it can be appreciated that the structure-based features matching algorithm used in this work presents a strong ability to eliminate incorrect matches, even with a very high percentage of outliers. This is also illustrated in Figure 8(b), where the evolution of the *precision* has been drawn. Similar to the *TruePos* value, the precision rapidly decreases for all the matching algorithms analyzed in this comparative study, being this decreasing less pronounced in the proposed structure-based features matching algorithm. These two graphs show the high performance of the weighted maximum clique strategy for solving matching problems. Figure 9(a–c) illustrate three visual examples of the proposed matching algorithm for 80% of outliers (results of the matching process proposed in this work for the images of the Figure 7(a–c), respectively).

On the other hand, computational load of the matching algorithm has been also tested using these same experiments. Figure 8(c) draws the time processing for the algorithm against the percentage of outliers (all the experiments in this section were executed in a 1.66 GHz Pentium PC computer with 1 Gb of RAM). As is noted in the figure, for low percentage of outliers, the performance of all the algorithms is similar, but they diverge when the percentage of outliers is incremented (up to 50%). From the Figure 8(c), it can be appreciated that the matching algorithm based on structure used in our visual odometry system provides the best time processing results.

### 3.3. Evaluation of the Visual Odometry Application

**Figure 10.**
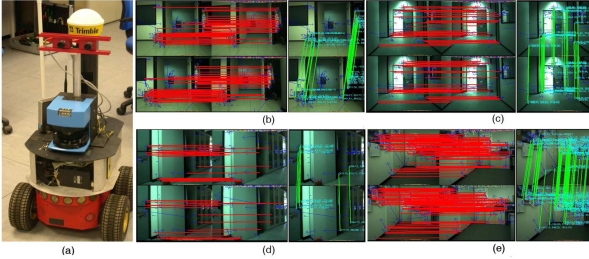
Activmedia P2AT robot used in the experiments. **(b–e)** four different image pair acquired by the stereo camera across the robot motion in the first test. Stereo and feature matching are shown in the figure (red and green lines, respectively).

To test the validity of the whole visual odometry system, we use an ActiveMedia Pioneer 2AT robot equipped with a stereoscopic camera (see Figure 10(a)) and a 1.66 GHz Pentium PC, equipped with a graphic processing unit NVIDIA 8800. The stereo head is the STH-MDCS from Videre Design, a compact, low-power color digital stereo head with an IEEE 1394 digital interface. The camera was mounted at the front and top of the vehicle at a constant orientation, looking forward. Images obtained were restricted to 320 × 240 pixels. Images were rectified before using the proposed approach.

Our robot was teleoperated through two different scenarios, indoor and outdoor, while capturing real-life stereo images. In each scenario, the robot followed different trajectories in order to compose a set of tests with which to evaluate the proposed visual odometry approach. Real tests for the indoor scenario are located at the research laboratories of the ISIS group in Málaga, a typical office-like environment where dynamic objects like persons were present. In this scenario, two different tests were achieved. On the other hand, real tests for the outdoor scenario are located at the campus of Teatinos at University of Málaga, a semi-structured environment with a high presence of people in the robot surrounding, and a sequence acquired by a stereo pair mounted on a moving car [25].

In the first test, the robotic platform starts in a room, is driven across a corridor and finishes its motion in a new room. The total distance traveled is about 40 m. In a similar experiment, the robot is teleoperated, and it moves from a room, across the corridor, closes a loop and finishes its motion in the same initial room. The total distance traveled in this test is about 80 m. The main novelty of this experiment is the presence of persons moving along the robot trajectory. On the other hand, in the test for the outdoor environment, the robot starts in the hall of the faculty, is driven across the faculty and it finish the motion, after closing a loop, in other place of the initial hall (the total distance traveled in this test is about 150 m). People and dynamic objects are highly present in this scenario. For each test, the experiment have been repeated 10 times trying to drive the robot by a similar path until the end of its motion.

**Figure 11.**
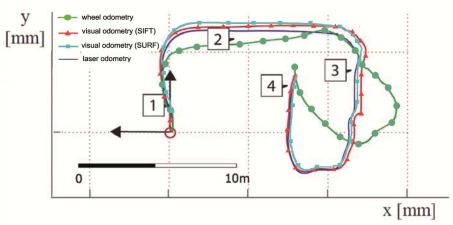
Trajectories estimated by visual (Harris, SIFT and SURF) and wheel odometry (black, red, cyan and green line, respectively) for the first test. Blue lines define the trajectory estimated by the laser scan matching. Robot poses at the captured times shown in Figure 10 are labeled.

The experimental results have been focused on the accuracy of the proposed algorithm. For all the experiments at the University of Málaga, the robot motion starts in the pose $p_{r}^{t}={\left(0,0,0^{{}^{\circ}}\right)}^{T}$ and it was teleoperated across the environment. In the Figure 10(b–e), we have illustrated four different captures from this real environment. Each image in the figure represents the stereo pair at two consecutive frames, top and bottom of the image, and the images used for the feature matching process (right image). The stereo matching and the feature matching is shown with red and green lines, respectively). The wheel odometry is also saved and compared to the visual odometry using Harris, SIFT or SURF features, and the results are also compared to the estimate of the robot trajectory using the results of the scan matching algorithm proposed by the authors [26]. This last algorithm was demonstrated to be an accurate and robust method for estimating the robot trajectory. We consider this laser odometry the ground truth of the robot motion (*i.e.*, statistical evaluation of our method is calculated using the results of the scan matching algorithm, which error was demonstrate to be lower than 1.2% and 0.8% for translation and rotation motions, respectively). Figure 11 shows the trajectories estimated by the proposed algorithm (black, red and cyan line for Harris, SIFT and SURF features, respectively) for this first trial. The wheel odometry (green line) and the trajectory estimated by the scan matching algorithm (blue line) are also drawn in the figure. Besides, the robot poses at the capture times shown in Figure 10(a–d) have been marked over this trajectory. As it is drawn in the figure, the visual odometry obtains an reliable estimate of the robot displacement, more similar to the trajectory estimated by the scan matching algorithm, and improving the internal odometry at the end of the experiment. There are small differences between the visual odometry obtained using SIFT, SURF or Harris corners, but the final error is similar.

**Figure 12.**
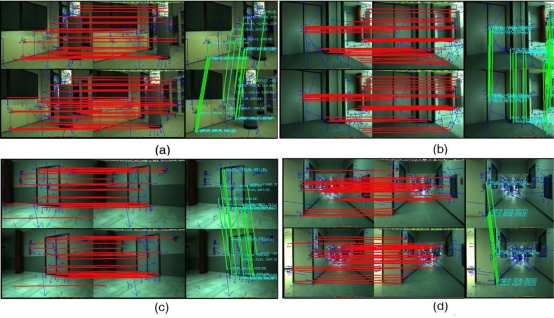
**(a–d)** Four different image pairs acquired by the stereo camera across the robot motion in the second reported trial. Stereo and feature matching are shown in the figure (red and green line, respectively).

**Figure 13.**
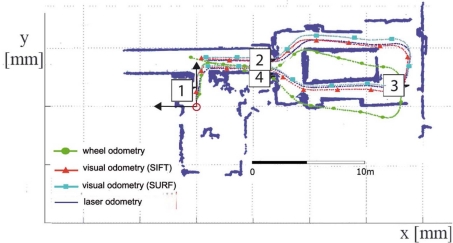
Trajectories estimated by visual (Harris, SIFT and SURF) and wheel odometry (black, red, cyan and green lines, respectively) for the second reported test. Blue line defines the trajectory estimated by the laser scan matching. Blue dots represent the map obtained using the scan data acquired by the laser range finder. Robot poses at the captured times marked over Figure 12.

For the second trial, the final location estimate by the proposed algorithm was, for Harris, SIFT and SURF, respectively, (3, 752*mm*, −210*mm*, −89.45°)*^T^*, (4, 340*mm*, −135*mm*, −92.15°)*^T^* and (4, 410*mm*, −143*mm*, −92.0°)*^T^*, while the odometry estimate by the wheel odometry was (3, 484*mm*, −1, 392*mm*, 66.15°)*^T^*. In Figure 12(a–d), four different stereo captures from this second real environment have been included, similar to Figure 10, where the stereo matching results are represented by red and green color, respectively. The trajectories estimated by the visual odometry algorithm proposed in this work, by the robot wheel odometry and by the scan matching algorithm have been shown in Figure 13 (the robot poses at the capture times shown in Figure 12 is also marked over this figure).

**Figure 14.**
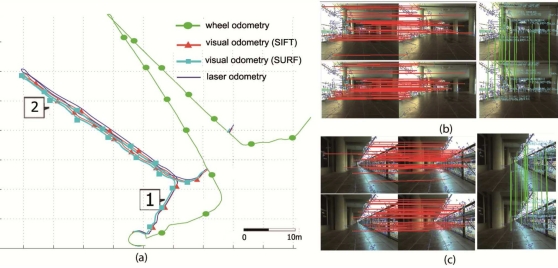
**(a)** Trajectories estimated by visual and wheel odometry (black, red, cyan and green line, respectively) for the third test (outdoor scenario). Blue lines define the trajectory estimated by the laser scan matching; and **(b)**, **(c)** two captures from the stereo camera and the results of the both matching processes.

On the other hand, the results for the test in the outdoor scenario is shown in Figure 14(a) (*i.e.*, trajectories estimated by the visual odometry, wheel odometry and scan matching algorithms are drawn using black, green, red, cyan and blue colors, respectively). As is shown in the figure, the pose estimated by the wheel odometry differs from the pose estimated by both visual and scan matching algorithm. The wheel odometry accumulates a high error at the end of the robot motion. However, results from the proposed approach are very similar to the pose estimated by the scan matching algorithm. Figure 14(b, c) show two different captures from this real environment (the robot poses at the instant time of this capture are marked in Figure 14(a)).

Table 3 summarizes the results described in this section. The accuracy of the visual odometry in each test is indicated by the 2D root-mean-square distance (RMS) at the final robot pose, taking into account the estimate given by the scan matching algorithm. Results of these experiments demonstrate the accuracy of the visual odometry algorithm. The resulting error is less than 1.5% of the traveled distance, or lower if the used descriptors are SIFT or SURF. Besides, the time processing of the matching stages (less than 20 ms) allows the robot to use this algorithm for estimating the robot displacement between consecutive frames. As is shown in the results, the accuracy of the visual odometry based on Harris corner is slightly lower than SIFT or SURF features, but appropriate for this type of application. However, the improvement on the computational load is remarkable compared to SIFT or SURF descriptors. These results associated to the visual odometry based on corner-like image features can be improved using other type of descriptor more complex. (Videos of these and more experiments are available in the address: http://robolab.unex.es/videos/visualodometry).

We have evaluated the use of the SIFT descriptor in the proposed visual odometry algorithm when it is used on a vehicle, like a car, which moves at velocity higher than the previous robot. Thus, a sequence of 865 image pairs taken from a stereo camera mounted on a moving vehicle has been used. This sequence is available on [25]. The acquisition device is a Videre Design MEGA-D stereo camera pair installed near the rearview mirror. The sequence is 15 fps, 320 × 240, color. The ground-truth of the motion is not included in the dataset. Besides, there is not loop-closing. Thus, it is not possible to obtain statistical information about the experiments. We have only evaluated the number of false positives and true positives detected in the stereo images. For the entire sequence, we have aleatory selected 50 frames at the instant time *t* and the next frame (*i.e.*, at the instant time *t* + 1). For each pair, the number of false positives and true positives has been evaluated respect to the total number of correspondences. The percentage of true positives was high, (96%–98%), and we obtains low values of false positives (0.2%–0.4%).

Finally, in order to validate our results, the robot was moved in a closed loop on a typical indoor environment (the same used in previous experiments) over 30 m, and used the error in start and end poses. Table 4 compares this error for vehicle odometry and visual odometry (using different features) for five loops.

## 4. Conclusions and Future Work

This paper has presented a new approach to solve the visual odometry problem. The main novelty of this proposal is that the matching stage has been conducted by means of a structural matching which combines absolute and relative feature constraints in two consecutive stages. The first stage solves the stereo matching problem and returns a set of natural landmarks characterized by their features descriptors and their 3D positions on the camera coordinate system. Then, the second stage matches the sets of natural landmarks detected at two consecutive instants of time (*i.e.*, frames). The set of matchings provided by this second stage allows to find an estimate of the robot displacement between both frames. Both stages obtain the set of accepted matchings taken into account the structural configuration of the involved features. This is implemented at both stages using a graph approach: given the consistency matrix which stores all pairwise combinations of matchings between the two set of features, this matrix is considered as an adjacency matrix and then the set of mutually consistent matchings with the large weight is computed. This maximum-weight clique is found using a fast algorithm based on the classic branch and bound strategy. This algorithm employs a heuristic vertex-coloring to implement the pruning criteria [16] and a backtracking search by color classes [17]. Experimental results demonstrate the accuracy and robustness of the matching stage and the visual odometry algorithm for different detectors and descriptors.

Future work will be focused on the integration of all steps into programmable logic devices such as FPGAs, in order to reduce the computational time. The GPU could be also employed to solve other tasks different from the SIFT or SURF detection and description. With respect to the theoretical aspects, the algorithm for the maximum-weight clique problem could be compared to other approaches such as the ones that formulate the problem as a continuous quadratic optimization problem with simplex constraints [27]. Other features can be also tested.

## Figures and Tables

**Table 1.** Estimation of parameters for the visual odometry algorithm.

**Parameter**	**Stereo matching SIFT (SURF)**	**Parameter**	**Feature matching SIFT (SURF)**
$U_{T}^{t}$	200 (150)	$U_{T}^{f}$	200 (150)
$U_{R}^{t}$	0.5 (0.5)	$U_{R}^{f}$	100 (100)

**Table 2.** Performance of the matching algorithms used in the comparative study for various percentage of outliers.

**Algorithm**	**Statistical**	**Percentage of outliers [%]**				

		10	30	50	70	90

Weighted	True positive	0.945	0.921	0.916	0.813	0.687
	Precision	0.912	0.871	0.843	0.812	0.771
	Time processing (s)	0.011	0.024	0.098	0.145	0.321

Non-weighted	True positive	0.919	0.904	0.818	0.587	0.189
	Precision	0.900	0.861	0.811	0.525	0.231
	Time processing (s)	0.021	0.082	0.114	0.438	0.969

BBF	True positive	0.921	0.919	0.803	0.564	0.169
	Precision	0.879	0.801	0.717	0.561	0.220
	Time processing (s)	0.081	0.102	0.377	0.691	1.141

RANSAC + epipolar	True positive	0.951	0.948	0.912	0.781	0.521
	Precision	0.952	0.947	0.829	0.711	0.328
	Time processing (s)	0.010	0.018	0.111	0.599	1.990

**Table 3.** Evaluation of the algorithm for real experiments in indoor and outdoor environments (average values).

				**Visual odometry SIFT (SURF) [Harris]**		**Dead reckoning**	

Run	Distance (m)	Frames	Average time (ms)	2D RMS error	%	2D RMS error	%

Indoor							
1	41.3	615	14.4	0.16 m (0.26 m) [0.62 m]	0.38% (0.6%) [1.5%]	1.67 m	4.05%
2	79.12	1018	17.2	0.61 m (0.54 m) [1.2m]	0.77% (0.68%) [1.5%]	2.12 m	2.67%

Outdoor							
1	148.66	2508	20.7	0.88 m (0.85 m) [1.34 m]	0.59% (0.58%) [0.9%]	12.1 m	8.1%

**Table 4.** Loop closure error in percentage.

Run Number	1	2	3	4	5
Distance (m)	30.2	62.30	95.0	128.5	155.2

Dead reckoning	2.25%	11.25%	21.5%	33.0%	51.25%
SIFT descriptor	0.70%	1.2%	0.9%	1.1%	1.2%
SURF descriptor	0.75%	1.1%	1.8%	1.5%	1.7%
Harris corners	1.2%	1.4%	1.7%	1.5%	2.1%
